# System Vaccinology for the Evaluation of Influenza Vaccine Safety by Multiplex Gene Detection of Novel Biomarkers in a Preclinical Study and Batch Release Test

**DOI:** 10.1371/journal.pone.0101835

**Published:** 2014-07-10

**Authors:** Takuo Mizukami, Haruka Momose, Madoka Kuramitsu, Kazuya Takizawa, Kumiko Araki, Keiko Furuhata, Ken J. Ishii, Isao Hamaguchi, Kazunari Yamaguchi

**Affiliations:** 1 Laboratory of Blood and Vaccine safety, Department of Safety Research on Blood and Biologicals, National Institute of Infectious Diseases, Tokyo, Japan; 2 Laboratory of Adjuvant Innovation, National Institute of Biomedical Innovation (NIBIO), Osaka, Japan; 3 Laboratory of Vaccine Science, WPI Immunology Frontier Research Center (WPI-IFREC), Osaka University, Osaka, Japan; Georgia State University, United States of America

## Abstract

Vaccines are beneficial and universal tools to prevent infectious disease. Thus, safety of vaccines is strictly evaluated in the preclinical phase of trials and every vaccine batch must be tested by the National Control Laboratories according to the guidelines published by each country. Despite many vaccine production platforms and methods, animal testing for safety evaluation is unchanged thus far. We recently developed a systems biological approach to vaccine safety evaluation where identification of specific biomarkers in a rat pre-clinical study evaluated the safety of vaccines for pandemic H5N1 influenza including *Irf7, Lgals9, Lgalsbp3, Cxcl11, Timp1, Tap2, Psmb9, Psme1, Tapbp, C2, Csf1, Mx2, Zbp1, Ifrd1, Trafd1, Cxcl9, β2m, Npc1, Ngfr* and *Ifi47*. The current study evaluated whether these 20 biomarkers could evaluate the safety, batch-to-batch and manufacturer-to-manufacturer consistency of seasonal trivalent influenza vaccine using a multiplex gene detection system. When we evaluated the influenza HA vaccine (HAv) from four different manufactures, the biomarker analysis correlated to findings from conventional animal use tests, such as abnormal toxicity test. In addition, sensitivity of toxicity detection and differences in HAvs were higher and more accurate than with conventional methods. Despite a slight decrease in body weight caused by HAv from manufacturer B that was not statistically significant, our results suggest that HAv from manufacturer B is significantly different than the other HAvs tested with regard to *Lgals3bp*, *Tapbp*, *Lgals9*, *Irf7* and *C2* gene expression in rat lungs. Using the biomarkers confirmed in this study, we predicted batch-to-batch consistency and safety of influenza vaccines within 2 days compared with the conventional safety test, which takes longer. These biomarkers will facilitate the future development of new influenza vaccines and provide an opportunity to develop *in vitro* methods of evaluating batch-to-batch consistency and vaccine safety as an alternative to animal testing.

## Introduction

Vaccination is a beneficial and universal tool to prevent infectious disease [1]. Because most vaccines are derived from inactivated virus, bacteria or toxoids, contamination by incomplete inactivation can cause serious adverse events. Thus, historically, the safety of vaccines is strictly regulated by law and each batch of vaccine must be tested by the National Control Laboratories according to the guidelines published in each country, *e.g.* the European Pharmacopeia, United States Pharmacopeia and World Health Organization guidelines [2]. After the diphtheria toxoid (DT) immunization incident in Japan in 1950 that caused the death of 68 children and illness in over 600 infants owing to contamination by incomplete inactivation of DT [3], the abnormal toxicity test (ATT) (also known as general safety test) was introduced to the Japanese guidelines. This stated that the minimum requirement of biological products (MRBP) and all inactivated vaccines and toxoids was mandatory safety evaluation by ATT and other specific toxicity tests.

Influenza vaccine is one of the most widely used commercially available vaccines worldwide for preventing seasonal influenza and its complications. Influenza virus vaccine is mainly produced using embryonated fertilized chicken eggs and inactivated with formaldehyde. Whole particle influenza virus vaccine [WPv] was first licensed as an influenza vaccine in the US in 1945 [4] and is still used in some countries. Although WPv contains all the components of the influenza virus and induces strong immunity in the vaccinated individual, a high incidence of adverse events, including local reactions at the site of injection and febrile illness, particularly among children have been reported [5], [6]. Thus, most recent vaccines manufactured since the 1970s have been subvirion vaccines. The subvirion influenza HA vaccine [HAv] showed a marked reduction of pyrogenicity compared with WPv [7]. The trivalent influenza vaccine [TIV] is a recently developed subvirion influenza vaccine with components selected and updated each year to protect against one of the three main groups of circulating influenza virus strains in humans. TIV may be administered every year. Vaccine adjuvant, e.g. alum, MF59 and AS03, was also used to enhance immunity in preparation for the H5N1 pandemic [8]. To improve immunogenicity and reduce toxicity in addition to batch-to-batch quality assurance of influenza vaccine, seed lot systems, recombinant DNA technology, as well as animal and insect cell culture inactivated vaccine production systems were introduced. Despite the increase in many vaccine production platforms, adjuvants, additives and vaccine types, safety evaluation tests in the preclinical phase and batch release have been unchanged in most countries, including in Japan.

We previously reported that improved ATT could evaluate and assure the batch-to-batch consistency of vaccines more strictly compared with conventional methods [9]. In addition, we recently introduced a system biological approach to vaccine safety evaluation and demonstrated that specific biomarkers could be used to evaluate batch-to-batch consistency and safety of vaccines to diphtheria-pertussis-tetanus (DPT) [10], [11] and Japanese encephalitis virus (JEV) [12]. Most recently, we showed that a system biological approach could evaluate the safety of pandemic H5N1 influenza vaccine [13]. We found 20 biomarkers for the evaluation of batch-to-batch consistency and the safety of H5N1 vaccine compared with HAv.

In this study, we tested whether these biomarkers could evaluate batch-to-batch consistency and the safety of seasonal HAv, as well as adjuvanted whole virion-derived influenza vaccine, using a multiplex gene detection system. This method might facilitate the evaluation of batch-to-batch consistency of HAv and reduce the time required for batch release compared with conventional ATT. These biomarkers will help the future development of new *in vitro* methods to evaluate vaccine safety as an alternative to animal testing.

## Materials and Methods

### 1. Animals and Ethics statement

Eight-week-old male Fischer (F334/N) rats weighing 160–200 g were obtained from SLC (Tokyo, Japan). All animals were housed in rooms maintained at 23±1°C, with 50±10% relative humidity, and 12-h light/dark cycles for at least 1 week prior to the test use. All animal experiments were performed according to the guidelines of the Institutional Animal Care and Use Committee of the National Institute of Infectious Diseases (NIID), Tokyo, Japan. The study was approved by the Institutional Animal Care and Use Committee of NIID.

### 2. Vaccines

The following vaccines were used in this study: (1) PDv: inactivated monovalent A/H5N1 whole-virion influenza vaccine (derived from NIBRG-14: A/Vietnam/1194/2004) adjuvanted with aluminum hydroxide, containing 30 µg HA/ml; (2) WPv: inactivated whole trivalent influenza vaccine (A/Newcaledonia/20/99 (H1N1), A/Hiroshima/52/2005 (H3N2), and B/Malaysia/2506/2004); HAV: trivalent HA influenza vaccine (A/Solomon Island/3/2006 (H1N1), A/Hiroshima/52/2005 (H3N2), and B/Malaysia/2506/2004), containing 30 µg HA/ml each strain. For evaluation of commercially distributed HAV in Japan, we used trivalent HA influenza vaccine (A/Solomon Island/3/2006 (H1N1), A/Hiroshima/52/2005 (H3N2) and B/Malaysia/2506/2004), containing 30 µg HA/ml per strain. PDv and WPv were produced, and manufactured by the Chemo-Sero-Therapeutic Research Institute, Kaketsuken (Kumamoto, Japan). Licensed and authorized HAvs were purchased from four different manufacturers [HAv (Lot L03A) from Kaketsuken (Kumamoto), HAv (Lot 309) from Kitasato Institute (Saitama), HAv (Lot 343-A) from Denka Seiken Co., Ltd. (Tokyo), HAv (Lot HA082D) from Biken (Kagawa)] in Japan. All vaccines complied with the MRBP in Japan. HAv used in this study was tested and authorized by NCL (National Control Laboratory) for distribution in Japan.

### 3. Abnormal toxicity test

ATT was performed according to the MRBP [http://www.nih.go.jp/niid/en/mrbp-e.html] using rats with a slight modification. Each 5 ml of vaccine was intra-peritoneally (*i.p.*) injected into rats. Five milliliters of saline (SA) (Otsuka normal saline; Otsuka Pharmaceutical Factory Inc., Naruto, Tokushima, Japan) was *i.p.* injected as a control. One day after the injection, rat body weight was measured and peripheral blood was collected. The number of white blood cells was counted with a hemocytometer (Nihon Kohden, Japan).

### 4. RNA preparation

One day after injection, rats were sacrificed to obtain whole lung tissues. Organs were immediately frozen in liquid nitrogen for storage. Thawed tissue was homogenized and mixed with an Isogen reagent (Nippon Gene, Tokyo, Japan). Total RNA was prepared from the lysate in accordance with the manufacturer’s instructions. Poly (A)+ RNA was prepared from total RNA with a Poly (A) Purist Kit (Ambion, Austin, TX), according to the manufacturer’s instructions.

### 5. Quantitative RT-PCR analysis

Poly (A)+ RNA was used to synthesize first-strand cDNA using a First-strand cDNA Synthesis Kit (Life Science Inc., St. Petersburg, FL), according to the manufacturer’s instructions. Expression levels of biomarkers (**Table 1**) were analyzed by real-time polymerase chain reaction (PCR) using a 7500 Fast Real-Time PCR System (Applied Biosystems, Foster City, CA) with 7500 Fast System SDS Software Version 1.3. cDNA was amplified for real-time PCR using SYBR Green I (Molecular Probes Inc.) to detect the PCR products. One microliter of 6-fold diluted cDNA was used in a 20-µl final volume reaction containing 10 µl SYBR Green PCR Master Mix (Applied Biosystems), and forward and reverse primers were as described previously [13]. The 7500 Fast System was programmed to run an initial polymerase activation step at 95°C for 10 min followed by 40 cycles of denaturation (95°C for 15 s) and extension (60°C for 1 min). Product synthesis was monitored at the end of the extension step of each cycle. Gene expression values were normalized against rat GAPDH.

**Table 1:** Biomarkers to evaluate influenza vaccine safety.

Official Symbol	Official Full Name	Gene ID
*Irf7*	Interferon regulatory factor 7	293624
*Lgals9*	Lectin, galactoside-binding, soluble, 9	25476
*Lgalsbp3*	Lectin, galactoside-binding, soluble, 3 binding protein	245955
*Cxcl11*	Chemokine (C-X-C motif) ligand 11	305236
*Timp1*	TIMP metallopeptidase inhibitor 1	116510
*Tap2*	Transporter 2, ATP-binding cassette, sub-family B	24812
*Psmb9*	Proteasome (prosome, macropain) subunit, beta type, 9	24967
*Psme1*	Proteasome (prosome, macropain) activator subunit 1	29630
*Tapbp*	TAP binding protein (tapasin)	25217
*C2*	Complement component 2	24231
*Csf1*	Colony stimulating factor 1 (macrophage)	78965
*Mx2*	Myxovirus (influenza virus) resistance 2	286918
*Zbp1*	Z-DNA binding protein 1	171091
*Ifrd1*	Interferon-related developmental regulator 1	29596
*Trafd1*	TRAF type zinc finger domain containing 1	114635
*Cxcl9*	Chemokine (C-X-C motif) ligand 9	246759
*β2m*	Beta-2 microglobulin	24223
*Npc1*	Niemann-Pick disease, type C1	266732
*Ngfr*	Nerve growth factor receptor	24596
*Ifi47*	Interferon gamma inducible protein 47	246208

### 6. QuantiGene Plex assays

QuantiGene Plex (QGP) assays were performed according to the QuantiGene Plex Reagent System instructions (Panomics Inc., Fremont, CA), as described previously [11]. Briefly, 10 µl of starting poly (A)+RNA (50 ng) was incubated for 10 min at 65°C, then mixed with 33.3 µl of lysis mixture, 40 µl of capture buffer, 2 µl of capture beads, and 2 µl of the target gene-specific probe set. Probe sets were heated for 5 min prior to use. Each sample mixture was then dispensed into an individual well of a capture plate, sealed with foil tape and incubated at 54°C for 16–20 h. The hybridization mixture was transferred to a filter plate, and the wells were washed three times with 200 µl of wash buffer. Signals for the bound target mRNA were developed by sequential hybridization with branched DNA (bDNA) amplifier, and biotin-conjugated label probe, at 48°C for 1 h each. Two washes with wash buffer were used to remove unbound material after each hybridization step. Streptavidin-conjugated phycoerythrin was added to the wells and incubated at room temperature for 30 min. The luminescence of each well was measured using a Luminex 100 microtiter plate luminometer (Luminex). Two replicate assays measuring RNA directly (independent sampling n = 6 for mRNA, n = 3–5 for lysate) were performed for all described experiments. The 20 target genes and GAPDH mRNA were quantified, and the ratio of the target genes to GAPDH mRNA was calculated.

### 7. Statistical analysis

Multiple comparisons were performed for SA, PDv, WPv and HA. To determine differences between manufacturers, multiple comparisons were performed for SA and HA from manufacturers A, B, C and D. Statistical analysis was performed in GraphPad Prism 6 (GraphPad Software, La Jolla, CA) using an ordinary one-way analysis of variance test followed by a Tukey multiple comparison test.

## Results

### Optimization of multiple gene detection system, QuantiGene Plex, for safety evaluation of the influenza vaccine

**Figure 1:**
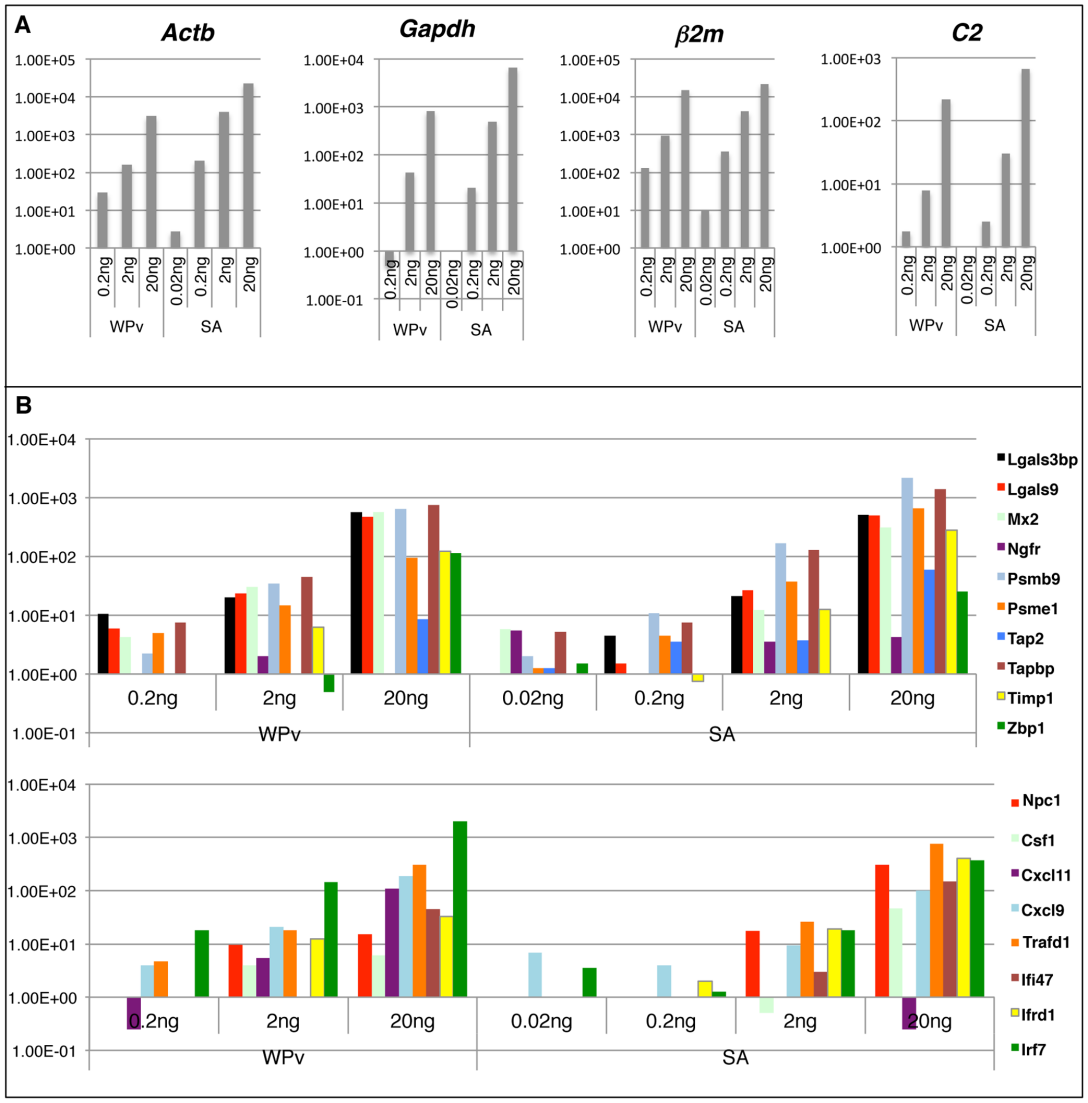
Optimization of QGP in influenza vaccine safety evaluation. A) Gene expression of *Actb*, *Gapdh*, *B2m* and *C2* and B) biomarkers, in 0.2, 2 and 20 ng RNA-containing samples from SA- and WPv-treated rat lungs. Relative expression levels of the *Gapdh* gene are indicated. SA: saline, WPv: Whole particle virion influenza vaccine.

We previously reported that 20 selected genes (**Table 1**), from 76 differentially expressed genes in adsorbed PDv-treated rats, could be used as biomarkers to evaluate H5N1 influenza vaccine safety compared with other types of influenza vaccine using conventional real-time PCR [13]. To establish faster and more convenient methods to detect these biomarkers in one-step as a new vaccine safety test, we used QuantiGene Plex (QGP) technology (Panomics Inc., Fremont, CA). We designed a custom QGP 2.0 assay to enable the measurement of expression levels of identified biomarkers. The Panomics QGP 2.0 assays provided quantitative measurements of 3 to 80 target RNAs per well by using bDNA technology in conjunction with multi-analyte magnetic beads to provide the detection and quantitation of multiple mRNA targets simultaneously. bDNA technology is a hybridization-based methodology that uses labeled DNA probes to amplify the signal rather than the target mRNA. Here, we produced probes for 20 genes and two control genes (*Actb* and *Gapdh*) for the one-step detection and quantification of these biomarkers. To check the sensitivity of probes and dynamic range of our biomarkers, we prepared 0.02, 0.2, 2 and 20 ng total RNA samples from WPv and SA-treated rat lungs and performed QGP analysis. Two control genes and two biomarkers (*β2m* and *C2*) reacted in a dose-dependent manner (**Figure 1A**). We re-evaluated all probes with the same sample. Each biomarker reacted in a dose-dependent manner (**Figure 1B**) except *Ngfr* and *Npc1*. Therefore, 20 ng of RNA sample was used for multiplex gene detection. All biomarkers except *β2m* reacted in a dose-dependent manner. *β2m* was saturated when using 20 ng RNA sample; thus *β2m* could not be used for QGP analysis.

### Validation of QGP with real-time PCR

**Figure 2:**
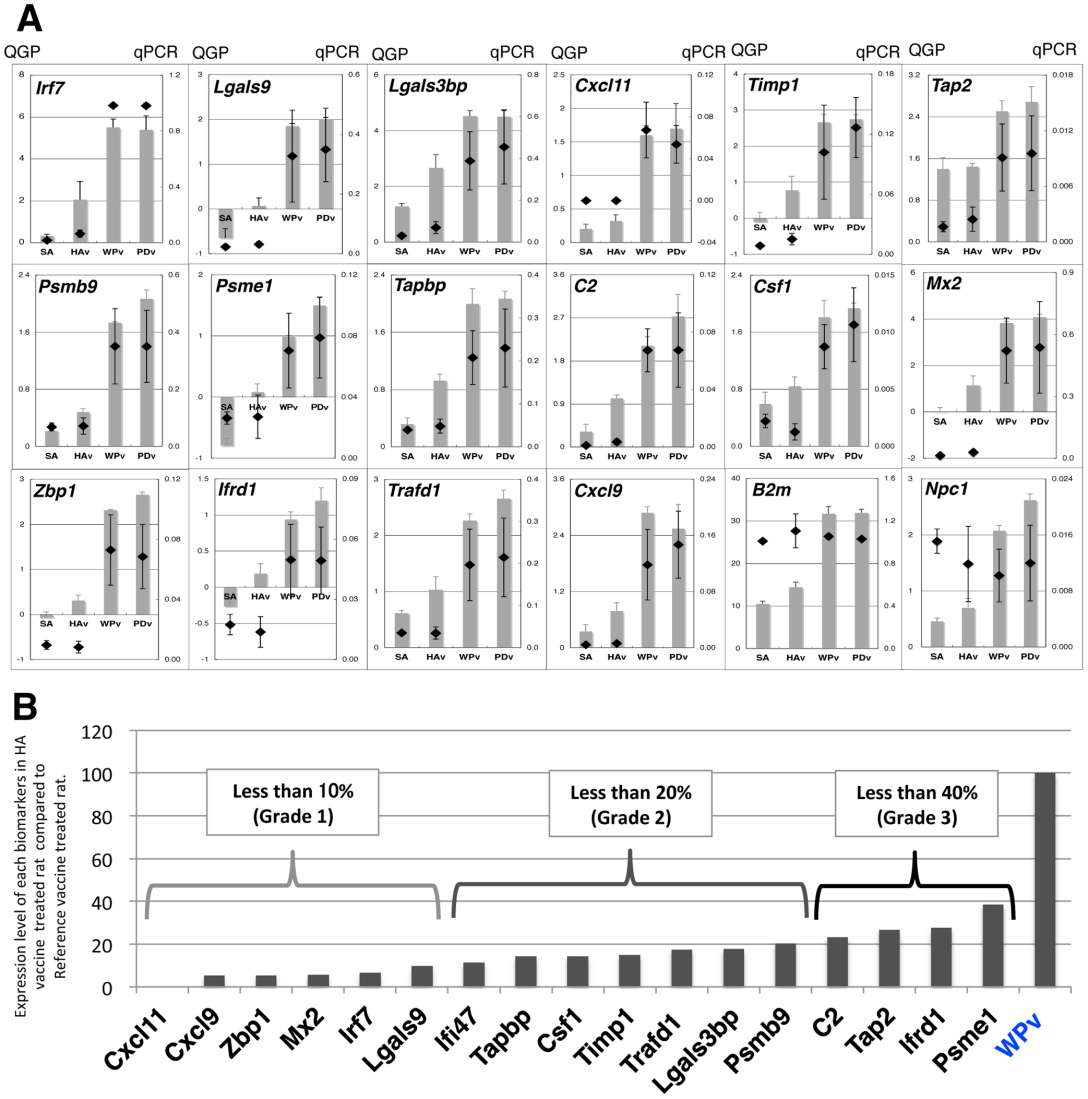
Validation of QGP with real-time PCR methods. A) GQP result was validated with real-time PCR methods. Bar graph indicates the real-time PCR results and dot blot indicates QGP results. B) Biomarkers were classified into three grades according to the relative expression level compared with WPv-treated rats.

To validate QGP, we performed real-time PCR analysis using the same samples. As a result, most biomarker gene expression data from the QGP correlated with the real-time PCR result except for *β2m*, *Npc1* (Figure 2) and *Ngfr* (data not shown). Finally, 17 genes were selected as the multiplex detection biomarker set. We next determined the relative biomarker expression levels in HAv-treated rat lungs compared with WPv used as a reference toxicity vaccine in the leukopenic toxicity test (LTT) in Japan. We classified *Cxcl11*, *Cxcl9*, *Zbp1*, *Mx2*, *Irf7* and *Lgals9* as a “Grade 1” gene set where relative expression levels in HAv compared with WPv were less than 10%. Likewise, we classified *Ifi47*, *Tapbp*, *Csf1*, *Timp1*, *Trafd1*, *Lgals3bp* and *Psmb9* as a “Grade 2” gene set where relative expression levels were less than 20% and *C2*, *Tap2*, *Ifrd1* and *Psme1* as a “Grade 3” gene set where relative expression levels were less than 40% in HAv compared with WPv. In Japan, it is acceptable for leukopenic toxicity levels of HAv to be not more than 20% of WPv by LTT. We applied LTT criteria for selecting and subdividing these biomarkers into three grades with expression levels below 20% of WPv and others.

### Evaluation of HAv safety in Japan using ATT and QGP

**Figure 3:**
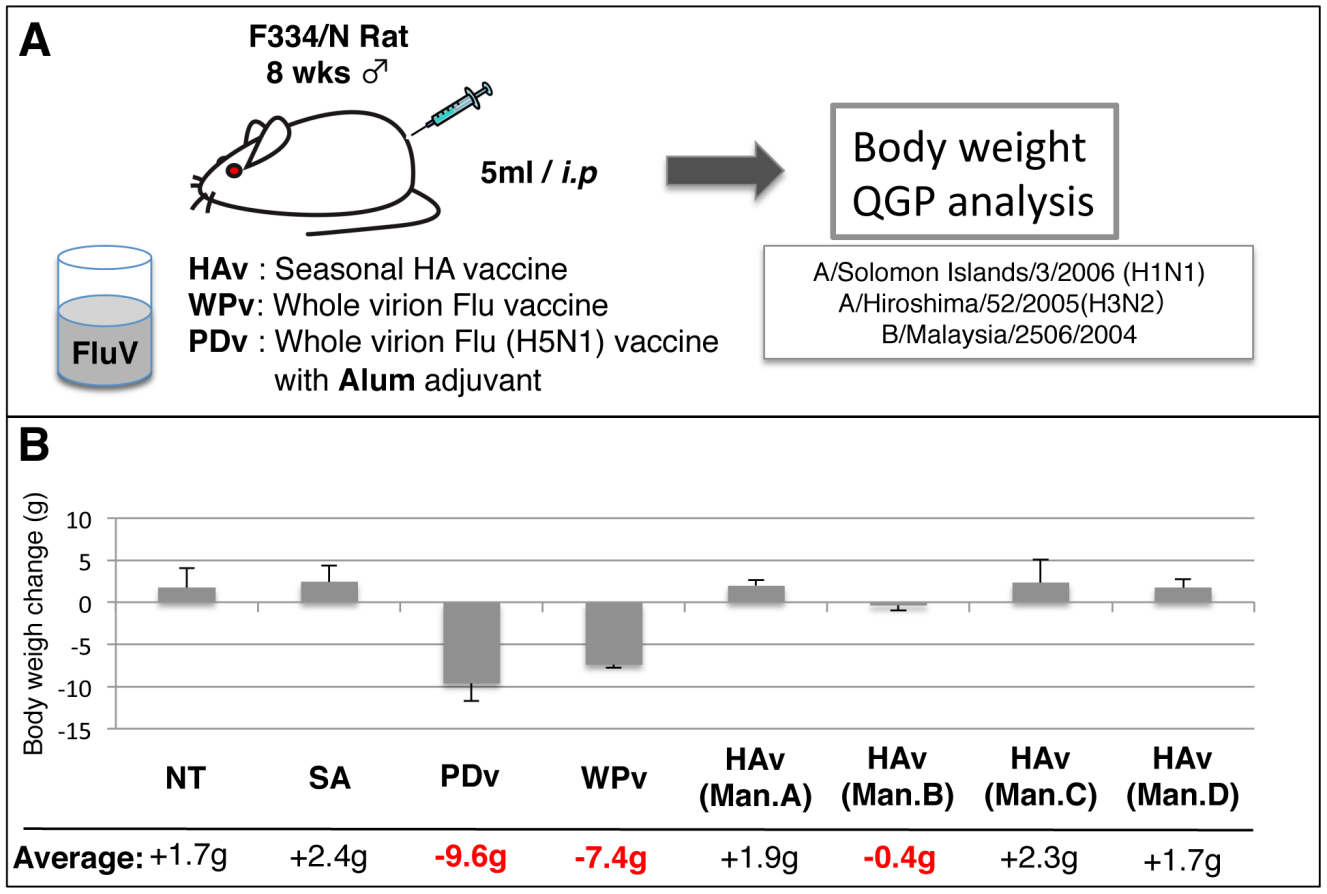
Evaluation of seasonal influenza vaccine with conventional animal safety test. A) The abnormal toxicity test was performed according to the Minimum Requirements of Biological Products. Each 5 ml vaccine was *i.p.* injected into rats, the body weight measured and lung tissues collected at day 1 after injection. B) Body weight change at day 1 after injection. NT: nontreated rat, SA: saline, PDv: pandemic H5N1 whole virion-derived vaccine with alum adjuvant, WPv: whole particle virion influenza vaccine, HAv: influenza HA vaccine, Man: manufacturer.

**Figure 4:**
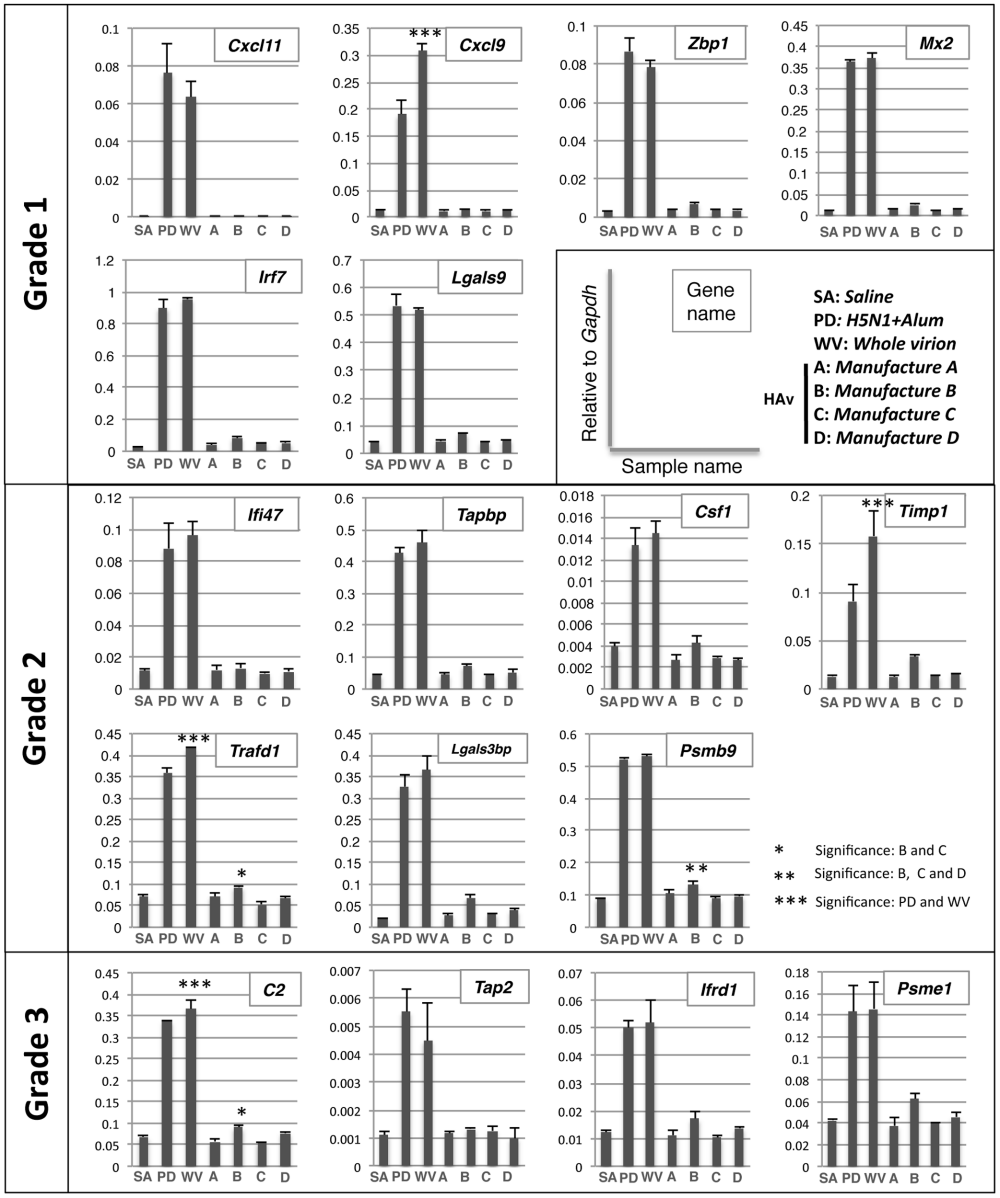
Evaluation of seasonal influenza vaccine with QGP. The relative gene expression levels of the *Gapdh* gene are indicated in each column (grades 1, 2 and 3, respectively). *Significant difference between B and C. **Significant difference between B, C and D, ***Significant difference between PD and WPv.

To evaluate the toxicity of seasonal HAv using biomarkers, we purchased market authorized seasonal influenza vaccines distributed in Japan from four different manufacturers (Kaketsuken, Denka Seiken, Kitasato, and Biken). Although the vaccines have been evaluated and passed ATT by the NCL according to the Japanese guidelines for MRBP, the reactogenicity of the vaccine to animals (rats, mice and guinea pigs) was varied. To evaluate these differences, we performed ATT and checked the body weight changes of rats after *i.p.* injection of each HAv (Figure 3A). Although treatment with PDv or WPv (toxic reference whole virion-derived vaccines) significantly decreased the body weight of rats, HAvs from three different manufacturers had no effect on body weight. HAv from manufacture B reduced the body weight of rats at day 1 (Figure 3B). However, there was no significant difference in rat body weight change for the other HAvs; thus HAv from manufacturer B might be slightly different, when comparing the mean body weight at day 1. In addition, there was no significant difference in leukocyte numbers following administration of HAv from the four manufacturers (data not shown). To evaluate the differences of each HAv, we next performed multiplex biomarker detection by QGP. No biomarkers were significantly up-regulated in HAv-treated rats compared with controls (Figure 4) except for *Psmb9*. Furthermore, *Psmb9* expression was significantly up-regulated following administration of HAv from manufacturer B compared with the control SA-treated and HAvs from the other manufacturers. The expression levels of *C2* and *Trafd1* were also significantly up-regulated in the HAv from manufacturer B compared with the HAv from manufacturer C.

### Biomarkers to evaluate safety of adjuvanted influenza vaccine

Both PDv and WPv contain the whole virion influenza vaccine and alum adjuvant is only added to PDv to enhance its immunogenicity. There was no difference in body weight change between WPv- and PDv-treated rats (Figure 3B). However, among the 17 biomarkers, the expression level of three genes, *Cxcl9*, *Timp1* and *Trafd1* in PDv-treated rats were significantly decreased compared with WPv-treated rats (Figure 4). Thus, these biomarkers could potentially evaluate the aluminum adjuvant effect.

### Cluster analysis of QGP data predicts influenza vaccine safety

**Figure 5:**
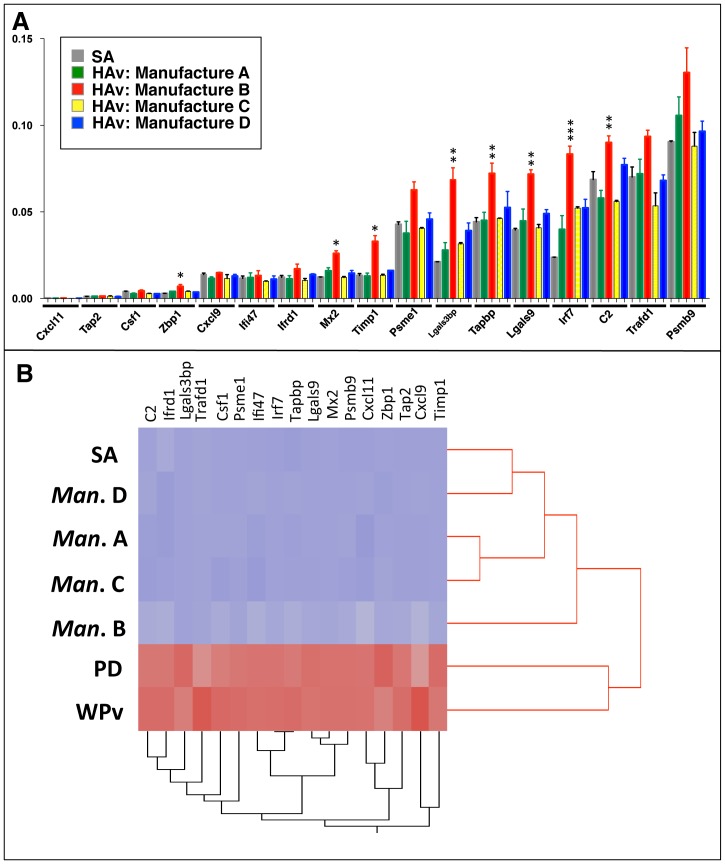
Evaluation of seasonal influenza vaccine with QGP and cluster analysis. A) Relative gene expression in HAv-treated rat lungs to *Gapdh* is indicated in the bar graph. B) Hierarchical clustering analysis with biomarkers could predict differences in HAv manufacturers as B is located in a separate cluster from other HAvs.

Conventional animal tests such as ATT and LTT have been performed in Japan for the evaluation of influenza vaccine safety and toxicity. Despite applying these tests that evaluate whole virion-derived influenza vaccine from HAv, it is difficult to distinguish statistically between different HAvs if they do not have comparable toxicity greater than 20–50% to WPv. According to the body weight change observed with ATT, we speculated that HAv from manufacturer B was slightly different than the others tested (Figure 3B), although this was not statistically significant. However, when biomarkers were used with QGP to evaluate HAvs, we could distinguish the HAv from manufacturer B compared with those from other manufacturers. When we focused on biomarker expression among the HAv-treated rat lungs, the expression levels of *Zbp1*, *MX2*, *Timp1*, *Lgals3bp*, *Tapbp*, *Lgals9*, *Irf7* and *C2* were significantly up-regulated in rat lungs treated with HAvs from manufacturer B (Figure 5A). In addition, cluster analysis with the biomarkers predicted differences in HAvs as the vaccine from manufacturer B was located in a separate cluster from the other HAvs. Thus, these biomarkers can evaluate batch-to-batch and manufacturer-to-manufacturer differences in HAvs (Figure 5B).

## Discussion

Vaccine safety is critical in the process of vaccine development and universal vaccination. Several vaccines were stopped owing to safety concerns, including severe side effects, after they had received marketing authorization and licensing, even when they were effective [14]. To ensure the safety of vaccines, the preclinical phase in the development of vaccines and the batch release system after marketing authorization is critical. However, the guidelines for nonclinical assessment of vaccines and batch release tests only focus on the evaluation of vaccine efficacy and immunogenicity in animal models, quality control testing programs and toxicology testing in relevant animal models [15]. These guidelines do not include scientific research for identifying the potential toxicities of the vaccines, adjuvants and additives.

We have demonstrated the advantage of a system biological approach using several vaccines authorized in Japan, *e.g.* DPT, JEV and Influenza vaccine including H5N1 pandemic influenza vaccine [10]–[13]. We successfully identified several biomarkers to evaluate DPT, JEV and influenza vaccine toxicity. In this study, we demonstrate that the biomarkers used to evaluate H5N1 pandemic influenza vaccine could also be used to evaluate the batch-to-batch consistency and the safety of HAvs. In addition, they can be used to evaluate manufacturer-to-manufacturer differences using the multiplex gene detection system. The biomarker analysis correlated to findings from conventional animal use tests, such as ATT. In addition, sensitivity of toxicity detection and differences in HAvs was higher and more accurate than with conventional methods. Despite all the HAvs evaluated in this study meeting MRBP criteria and passing NCL, our results suggest that HAv from manufacturer B is slightly different than the HAvs according to *Lgals3bp*, *Tapbp*, *Lgals9*, *Irf7* and *C2* gene expression. Among the official vaccine adverse event information provided by the Japanese authorities, there is no reported evidence that the adverse event rate was increased or that severe adverse events were observed caused by HAv from manufacturer B. It is still unknown what factors (additives, formalin content, protein content) induce these biomarkers in the HAv from manufacturer B. Further studies are needed to determine whether our biomarkers could predict the toxicity of influenza vaccine by using different formulations of HAv. Using biomarkers from any grade characterized in this study, we could also predict the safety of influenza vaccines within 2 days whereas the conventional animal use safety test, ATT requires 7 days for evaluating batch-to-batch consistency and vaccine safety. Further studies are needed to determine how these biomarkers can be used to evaluate the safety of HAv. To set the percent limit of up-regulation of each biomarker, it might be helpful to compare another conventional test such as LTT [[http://www.nih.go.jp/niid/en/mrbp-e.html]] as well as a comparison of failed batches of HAv. LTT evaluates the peripheral leukocyte number reduction rate compared with WPv. In general, WPv induces a strong loss of peripheral leukocyte numbers 16 hours after WPv administration in mice [9 and 28]. The test criteria of LTT is that the loss of leukocyte numbers in test samples must be no greater than 20% compared with a reference toxic vaccine such as WPv or less than 50% of SA-treated mice. These criteria may be applicable to set our biomarker expression limit. Further validation is required to set the limit the gene expression level.

**Figure 6:**
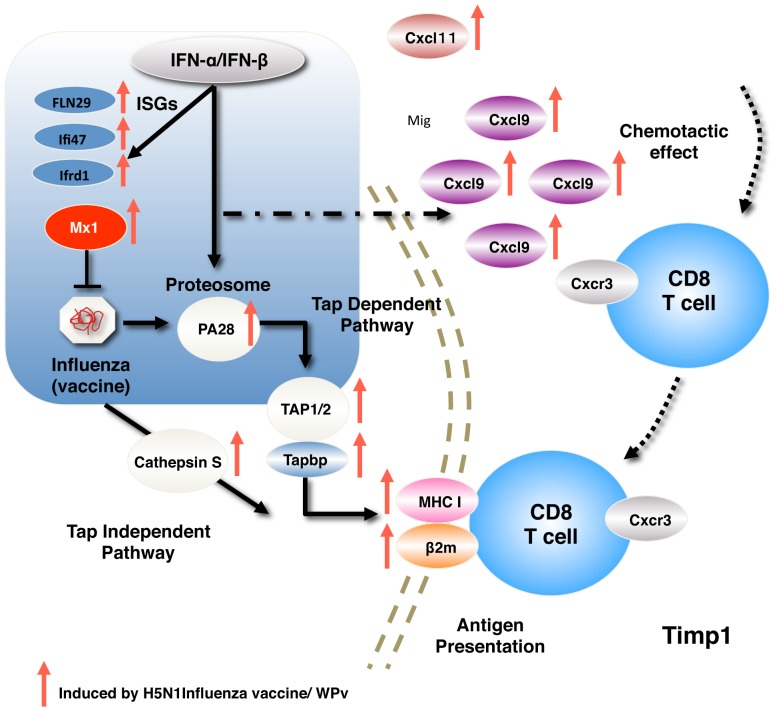
Summary of biomarker studies. Biomarkers used in this study were strongly correlated with immune responses after influenza infection.

Influenza is a socially important infectious disease that causes seasonal flu outbreaks worldwide and has a pandemic status [16]. Correspondingly, many types of influenza vaccine (cell derived, recombinant derived, live attenuated and inactivated influenza vaccine), have been developed to ensure efficacy and reduce toxicity [17]. While some adjuvants have been developed and used to amplify vaccine efficacy [8], the safety of adjuvants is still of concern. Recently, several adjuvants (squalene-based MF59 and AS03) developed and licensed for use only in pandemic influenza vaccines were under investigation for the occurrence of narcolepsy in vaccinated children in European countries [18]. Conventional safety tests could be used to evaluate the safety of these vaccines [19], but it is still difficult to predict the safety and toxicity of influenza vaccines, adjuvants and additives [20]. We demonstrated that usage of system biological approaches to evaluate safety might revolutionize vaccine testing methods [21]. Most of the previously identified biomarkers were up-regulated and correlated with influenza infection, interferon responses, antigen presentation and antibody production (Figure 6). In addition, we found that several biomarkers, *Cxcl9*, *Trafd1*, and *C2* were candidates for evaluating differences between alum-adjuvanted influenza vaccines and nonadjuvanted vaccines. Further studies, using several adjuvants, are needed to confirm the feasibility of these biomarkers in evaluating adjuvant safety.

In addition to whole transcriptome analysis of vaccinated animals, recent advances in genome research enabled the acquisition of whole transcriptional data from vaccinated individuals and identification of gene expression after immunization with vaccines to yellow fever, measles, tularemia and tuberculosis [22]. With a focus on the influenza vaccine, Bucasas et al. reported a 494 gene set, including biomarkers identified in our previous study (*MX1*, *IRF7*) that strongly correlated with antibody responses in humans [23]. Wei et al. reported gene expression differences between HAv and live attenuated influenza vaccine. They identified 265 differentially expressed genes, including our previously identified biomarkers, *IRF7*, *MX1*, *MX2*, *OAS1* and *ZBP1* [24].

**Figure 7:**
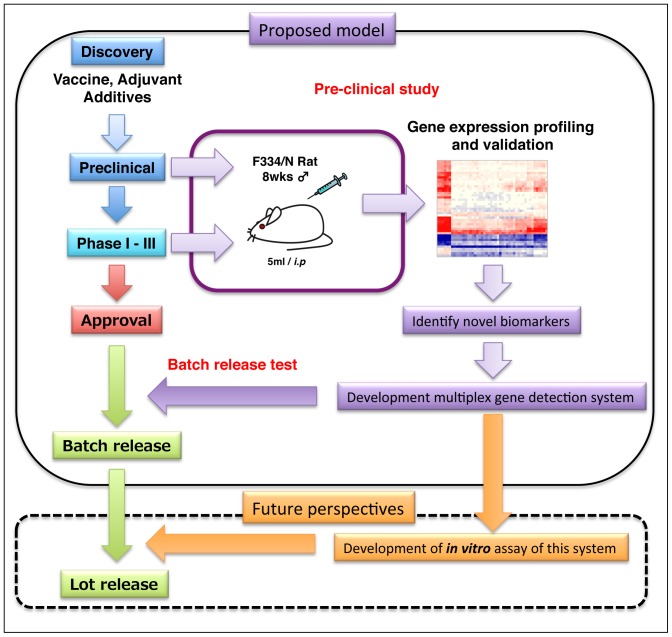
Application of the system biological approach for influenza vaccine development. Proposed model of future influenza vaccine development and establishment of preclinical studies and batch release testing. Acquisition of transcriptome data at the preclinical and clinical phase is useful for future batch release testing and the prediction of vaccine efficacy and toxicity.

Recently, Nakaya and Pulendran reported a system biological approach, termed systems vaccinology [25], which was used to predict immunogenicity and provide new mechanistic insights regarding influenza vaccination. They also reported several gene sets that predicted influenza vaccine immunogenicity, including our previously identified biomarkers, *MX1*, *MX2*, *OAS1* and *IRF7* [26]. More recently, Franco et al. reported 20 genes, including our biomarkers, *TAP2* and *OAS1*, which correlated with antibody responses, using integrative genomic analysis [27]. All these reports suggest that using animal models is still useful if biomarkers are up-regulated in vaccinated individuals and can reveal the role of biomarkers in immune responses and vaccination toxicity. Thus, in the preclinical and clinical phase, the acquisition of transcriptome data from both vaccinated individuals and animals, and a comparison of these data will be helpful for future vaccine development and batch release testing (Figure 7).

Taken together, system biological approaches to identify vaccine toxicity using whole genome transcriptome methods will improve vaccine development in preclinical and clinical phases if more data are generated from successfully vaccinated individuals and those with side effects. It is still unclear whether and how these factors determine immunogenicity and toxicity. Further studies are required to identify and reveal the mechanisms underlying vaccination in humans and in animal models, including nonhuman primates.
